# Integrin β4 promotes invasion and anoikis resistance of papillary thyroid carcinoma and is consistently overexpressed in lymphovascular tumor thrombus

**DOI:** 10.7150/jca.36125

**Published:** 2019-10-21

**Authors:** Jian Li, Minghua Luo, Huiting Ou, Xiaoling Liu, Xueling Kang, Weihua Yin

**Affiliations:** 1 Department of Pathology, Peking University Shenzhen Hospital, Shenzhen, Guangdong Province, 518036, China.; 2 State Key Laboratory of Chemical Oncogenomics, Peking University Shenzhen Graduate School, Shenzhen, Guangdong Province, 518055, China.; 3 Department of Endocrinology, Shenzhen Second People's Hospital, Guangdong Province, 518035, China.; 4 Department of Thyroid and Breast Surgery, Peking University Shenzhen Hospital, Shenzhen, Guangdong Province, 518036, China.; 5 Department of Oncology, Peking University Shenzhen Hospital, Shenzhen, Guangdong Province, 518036, China.

**Keywords:** integrin β4, papillary thyroid carcinoma, anoikis, lymphovascular tumor thrombus

## Abstract

Although the majority of papillary thyroid cancers (PTC) are indolent, a subset of PTCs behaves aggressively due to extensive invasion and distant metastasis. Integrin β4, a member of the integrin family, has been shown to enhance the progression in some malignancies; however, its role in PTC remains unclear. Here, we demonstrated that β4 overexpression was associated with extrathyroid extension, lymph node metastasis, high TNM stage, and poor overall survival based on The Cancer Genome Atlas cohort. Immunohistochemistry showed that β4 expression was significantly upregulated in the tumors with infiltrating growth pattern, as well as those with positive lymphovascular invasion. Moreover, β4 was invariably overexpressed in the lymphovascular tumor thrombi, which has not been reported before. After shRNA-induced knockdown of β4 *in vitro*, the migration, invasion and scratch repair ability of the tumor cells were significantly reduced. Furthermore, β4 reduction decreased anchorage-independent growth and increased anoikis. The bioinformatics analysis revealed that approximately 70 pathways were significantly dysregulated in the high β4 expression group. The MAPK pathway and propanoate metabolism were located in the network center of those pathways. Taken together, our results suggest that β4 could promote the tumor's aggressiveness by enhancing invasion and antagonizing anoikis. The upregulated expression of β4 in the tumor thrombi is intrinsically linked to its role in strengthening the anoikis resistance.

## Introduction

Invasion and metastasis are the main causes of death in malignancies 1-5. Papillary thyroid carcinoma (PTC) is generally considered a low-grade malignancy with an overall 5-year survival rate of nearly 95%. However, approximately 5-20% of PTCs show recurrence, and 10-15% present distant metastases, which exhibit enhanced aggressiveness 6-8. Despite the use of comprehensive treatments, including surgical resection, radioactive ^131^I, external radiation, and thyroid-stimulating hormone (TSH) inhibition, the mortality of highly aggressive PTC has not been significantly improved 9. Therefore, it is imperative to elucidate the potential mechanisms associated with highly invasive PTC and search for more effective therapeutic targets.

Integrin β4 is a member of the integrin family and forms a heterodimer with α6 subunit, which acts as an adhesion receptor for laminins in the extracellular matrix (ECM) 10. Under physiological conditions, β4 is mainly expressed on the basement membrane in epithelial cells and connects with the intracellular intermediate filaments and extracellular laminins to form hemidesmosomes. However, in the tumor microenvironment, β4 is mobilized from hemidesmosomes and moves to the filamentous actin protrusions, where it facilitates the migration of tumor cells 11, 12. In addition, β4 can function as a signaling transducer by activating pathways, e.g., PI3-K, MAPK, NF-κB, and thus promote tumor aggressiveness in some malignancies, such as squamous cell carcinoma, breast cancer, and gastric cancer 13-16. Thus β4 has been considered an attractive therapeutic target and a small molecular compound was recently developed to antagonize β4-overexpressed tumors 17-19.

Serini et al. first revealed the neoexpression of β4 by neoplastic cells in PTC in 1996 20. Since then, only one report further addressed the role of β4 in PTC and demonstrated the positive correlation between β4 expression and gross lymph node metastasis 21. To the best of our knowledge, no systematic evaluations have been conducted to illustrate the regulating function of β4 on the aggressiveness of PTC. To this end, we first investigated the relationship between β4 expression and clinicopathological features of PTC based on The Cancer Genome Atlas (TCGA) database; then the correlation of β4 expression with tumor's growth pattern, histological variant, BRAF^V600E^ mutation and lymphovascular invasion (LVI) based on immunohistochemical analysis; and finally the effects of β4 on the tumor cells' invasion, anchorage-independent growth, and anoikis based on shRNA-induced cell models *in vitro*. In addition, the metabolic and transductional pathways associated with high β4 expression were explored through *in silico* analysis.

## Materials and Methods

### TCGA data analysis

The clinical information and mRNA-seq data, including 507 thyroid carcinoma samples and 58 noncancerous thyroid tissues, were downloaded from TCGA database (https://portal.gdc.cancer.gov/) on February 25, 2018. All mRNA levels of the samples were normalized and measured using the IlluminaHiSeqRNA-seq V2 platform. Of the 507 cases of thyroid carcinoma, 505 were PTC, 1 was follicular thyroid carcinoma, and 1 was poorly differentiated thyroid carcinoma. There were 5 PTC samples without β4 mRNA expression data. Therefore, the number of PTC cases included in the study was 500. The median β4 mRNA level was set as the cut off value to stratify 500 cases of PTC into β4 mRNA high (>median value) and low (≤median value) expression groups. TNM classification of thyroid carcinoma provided by TCGA database was based on the sixth and seventh edition of the Cancer Staging Manual issued by American Joint Committee on Cancer (AJCC).

### Histopathologic category

PTC with a well circumscribed growth pattern (WC-PTC) was defined as that with an expansive growth pattern without a capsule or limited by a capsule with no or minimal invasion 22-24. WC-PTCs that were completely or almost entirely composed of follicles were considered as non-invasive follicular thyroid neoplasm with papillary-like nuclear features (NIFTP) 25, 26 and excluded from the research. Moreover, the presence of solid growth pattern, tumor necrosis, multifocal growth or increased mitotic activity (>5 per 10 high power fields) were also excluded from the analysis 22. PTC with a poorly circumscribed growth pattern (PC-PTC) was those that with an infiltrative growth pattern or with a widely invaded capsule 22, 24.

A total of 1008 consecutive cases of PTC resected at Peking University Shenzhen Hospital, Shenzhen, China, from January 1, 2016 to December 31, 2017 were enrolled. Two senior pathologists (Jian Li and Weihua Yin) reevaluated and confirmed the growth patterns and histological variants. The classification of histological variants was based on the 4th edition of the WHO classification of tumors of endocrine organs 27. Of the enrolled cases, there were 40 cases of WC-PTC, all of which were the classic variant of PTC, and 968 cases of PC-PTC, including 824 cases of the classic variant, 70 cases of the follicular variant, 31 cases of the oncocytic variant, 19 cases of the tall cell variant, 7 cases of the diffuse sclerosing variant, 12 cases of the solid variant, and 1 case of the cribriform-morular variant, and 4 cases of NIFTP. Twenty-five cases from the WC-PTC group, and 60 cases from the PC-PTC group, including 20 cases of classic variant, 20 cases of follicular variant and 20 cases of oncocytic variant were randomly selected. In addition, all of the following cases from the PC-PTC group, including 19 cases of tall cell variant, 7 cases of diffuse sclerosing variant and 12 cases of solid variant, were enrolled. Thus, the total number of cases studied was 123. The ratio of females to males was 84 to 39, and the mean patient age at surgery was 36.63±12.02 years (mean±SD). The clinicopathological data of the 123 patients were shown in Supplementary Table S1. The protocol and acquisition of tissue specimens in this study were reviewed and approved by the Ethics Committee of Peking University Shenzhen Hospital, and informed consent was provided by the patients involved.

### Immunohistochemistry

Four-micrometer-thick sections were processed by immunohistochemistry using an automated immunostainer (Ventana Benchmark® XT autostainer, Ventana Medical Systems, Inc., Tucson, AZ, USA). The sections were deparaffinized and rehydrated, and the endogenous peroxidase activity was blocked. Then, antigen retrieval was performed in citrate buffer for 24 min at 100°C. Next, the sections were incubated with a rabbit monoclonal anti-integrin β4 antibody (1:400 dilution, Cat. ab182120, Abcam, Cambridge, UK) for 24 min at 37°C. After the sections were washed with PBS, they were treated with an *ultraView* Universal HRP multimer (Ventana Medical Systems), which contained a cocktail of HRP-labeled goat anti-rabbit and anti-mouse secondary antibodies, for 8 min at 37°C, visualized with 3, 3′-diaminobenzidine chromogen (*ultraView* Universal DAB Detection kit, Ventana Medical Systems) and finally counterstained with hematoxylin. Negative controls were performed by omitting the primary antibody. The peripheral nerve inside the slide was used as an internal positive control.

### Evaluation on the β4 immunostaining score

Evaluation on β4 immunohistochemical staining was based on the methods introduced by Masugi et al. 28 and was modified as described below. Because the peripheral nerves exhibited consistent strong positive β4 staining, it was used as the internal positive control. The tumor cells of PTC demonstrated two distinct staining patterns: Pattern 1, equal or stronger diffuse cytoplasmic staining compared with that of peripheral nerves and Pattern 2, predominant basal membranous staining with weak or no cytoplasmic staining. Tumor cells with staining pattern 1 were considered β4-overexpressing cells. The percentage of β4-overexpressing cells per total tumor cells in each slide was assessed and scored as 1,2,3, or 4 if the percentage was <25% (one quarter), 25% to <33.33% (one third), 33.33% to <50% (one half), or ≥50%, respectively. The score was carefully evaluated by two senior pathologists (Jian Li and Weihua Yin). The histogram of the overexpression score was presented in Supplementary Figure S1 and the median score was 2. Tumors with scores of >2 were considered to have high β4 expression, and those with scores of ≤2 were defined to have low β4 expression.

### BRAF^V600E^ mutation detection by polymerase chain reaction (PCR) amplification

DNA was extracted from paraffin-embedded sections using the QIAamp DNA Mini Kit (Qiagen, Hilden, Germany) according to the manufacturer's instructions. The upstream and downstream primers targeting the BRAF exon 15 containing codon 600 were 5′- TCATAATGCTTGCTCTGATAGGA-3′ and 5′-GGCCAAAAATTTAATCAGTGGA-3′ as described previously 29. The primers were synthesized by Sangon Biotech Co., Ltd. (Shanghai, China). The A_260_/A_280_ values were between 1.8 and 2.0 in all DNA samples. A 25 μl reaction system was established for the PCR process, which included purified genomic DNA (0.1μg/μl) 2μl, forward primer (10μM) 0.5μl, reverse primer (10 μM) 0.5μl, 10 × buffer (Promega, Madison, WI, USA) 2.5μl, MgCl_2_ (25 mM) 1.5μl, dNTP (10mM) 1μl, Taq polymerase (Promega) 0.3μl (5U/μl), and the distilled water 16.7μl. The amplification protocol comprised 35 cycles: predenaturation at 95°C for 5 min; denaturation at 95°C for 30 sec; annealing at 55°C for 50 sec; extension at 72°C for 1 min; and a final extension at 72°C for 7 min. PCR products were electrophoresed on 2.5% agarose gels, purified and subsequently sent to Geneski Biotechnologies Inc. (Shanghai, China) for sequencing. The mutation status of the sequence was determined by comparison with the genomic DNA of BRAF.

### Cell lines

The thyroid cancer cell line K1 and the human primary thyroid epithelial cell line Nthy-ori 3-1 were obtained from the European Collection of Authenticated Cell Culture (ECACC, Salisbury, UK). The thyroid cancer cell lines BCPAP and TPC1 were obtained from the Cell Bank of the Chinese Academy of Science (Shanghai, China) and EK-biosciencs Co., Ltd. (Shanghai, China), respectively. All cells were cultured under standard conditions (37°C and 5% CO_2_). K1 and Nthy-ori 3-1 cells were grown in RPMI 1640 (Gibco, Carlsbad, CA, USA) supplemented with 10% FBS (Gibco) and 2 mM L-glutamine (Gibco). The BCPAP cells were cultured in RPMI1640 supplemented with 10% FBS. TPC1 cells were maintained in DMEM/F12 (Gibco) supplemented with 10% FBS.

### Knockdown of β4 expression by lentiviral short hairpin RNA (shRNA)

The recombinant lentivirus containing small interfering RNA targeting β4 (shRNA-β4) and a scramble negative control (shRNA-scramble) were commercially prepared by Genechem Co., Ltd. (Shanghai, China) 30. The targeting shRNA sequence for β4 was 5′-GAGGGTGTCATCACCATTGAA--3′, as reported previously 31. The scramble shRNA sequence was 5′-TTCTCCGAACGTGTCACGT-3′ 32. A lentivirus transfer vector (GV248) was ligated with the shRNA sequence. Lentiviral was packaged by transfection of 293T cells with the recombinant GV248, and 2 packaging vectors: pHelper 1.0 and pHelper 2.0. Three days after transfection, the lentivirus particles were collected, filtered, and concentrated by ultracentrifugation at 4°C from the supernatant of 293T cells. K1 cells were seeded in 12-well plates (3×10^4^ cells/well) and when grown to approximately 20% confluence, the cells were transfected with the lentiviral-shRNA at a final concentration of 5 pfu/cell for 16 h. Subsequently, the cells were changed to routine culture media and cultured continuously for 56 h. The green fluorescence of GFP in the infected K1 cells was observed by inverted fluorescence microscopy. If the fluorescence rate reached over 80%, the cells were collected for β4 knockdown efficiency examination and used for downstream experiments.

### Western blotting analysis

β4 protein expression was determined by Western blot analyses. The indicated cells were rinsed twice with ice-cold PBS and lysed in RIPA buffer containing protease inhibitors (Beyotime, Shanghai, China), and protein concentrations were measured by a BCA Protein Assay Kit (Beyotime). Twenty micrograms of protein from each sample was separated by 8% SDS-polyacrylamide gel electrophoresis (SDS-PAGE) and transferred onto polyvinylidene fluoride (PVDF) membranes (Thermo Fisher Scientific, Waltham, MA, USA). Membranes were incubated with a rabbit monoclonal anti-integrin β4 antibody (1:500 dilution, Cat.ab182120, Abcam) at 4°C overnight. The membranes were then washed three times with TBST buffer (0.05 mol/L Tris, 0.15 mol/L NaCl, and 0.05% Tween 20) and incubated with horseradish peroxidase conjugated goat anti-rabbit secondary antibody (1:2000 dilution, Cat.sc-2004, Santa Cruz, Dallas, TX, USA) for 1 h at room temperature. After three washes in TBST buffer, antibody binding was visualized using a Pierce™ ECL Western Blotting Substrate according to the manufacturer's protocol (Thermo Fisher Scientific). Experiments were repeated three times.

### Transwell migration assay

Cell migration was performed in transwell chambers (Corning Inc., Corning, NY, USA). K1 cells transfected with shRNA-β4 or shRNA-scramble (1×10^5^ cells/well) were seeded in the upper chambers with serum-free medium. The lower chambers contained 10% FBS medium as an attractant. After incubation at 37°C for 48 h, the migrated cells on the lower membrane surface were fixed in methanol and stained with crystal violet. The number of migrating cells in 9 randomly selected fields was counted under 200× microscope fields, and the means for each chamber were determined. Experiments were repeated three times.

### Transwell invasion assay

Cell invasion was performed in Corning® BioCoat™ Matrigel® Invasion Chamber (Corning Inc.). K1 cells transfected with shRNA-β4 or shRNA-scramble (1×10^5^ cells/well) were seeded in the upper chambers with serum-free medium, and the lower chambers contained 10% FBS medium as an attractant. After 72 h, the invasive cells on the lower membrane surface were fixed in methanol and stained with crystal violet. The number of invasive cells in 9 randomly selected fields was counted under 200× microscope fields, and the means were determined. Experiments were repeated three times.

### Wound healing assay

K1 cells transfected with shRNA-β4 and shRNA-scramble were seeded into a 96 Wounding Replicator (VP Scientific, San Diego, CA, USA) at a density of 5×10^4^ cells/well with medium containing 10% FBS and grown until the monolayer adherent cell confluence was >90%. A wound was scraped across the cell monolayer. Culture plates were washed with serum-free medium and incubated with medium containing 0.5% FBS. Photomicrographs were taken at the zero time point, 24h, and 48h to measure the ratio of the migrating distance relative to the initial wound distance. Experiments were repeated three times.

### Soft agar assay and anoikis analysis

K1 cells transfected with shRNA-β4 and shRNA-scramble were suspended in RPMI 1640 with 10% FBS containing 0.3% low melt agarose (Promega) and overlaid (3×10^3^ cells/well) on a solid 0.6% agar layer in six-well plates. The cultures were maintained for 14 days, and RPMI 1640 medium with 10% FBS was added during the process to keep the upper agarose moist. Then, the colonies containing 50 cells or more were quantified by counting 5 randomly selected fields per well under ×40 microscope fields.

Anoikis was assessed by measuring cytoplasmic DNA fragmentation using a Cellular DNA Fragmentation ELISA Kit (Roche Diagnostics, Mannheim, Germany) following the manufacturer's protocol. Briefly, K1 cells transfected with shRNA-β4 or shRNA-scramble (1×10^5^ cells/well) were seeded on an ultra-low attachment 24-well plate (Corning, Inc.) and incubated with 5'-bromo-2'-deoxy-uridine (BrdU) labeling solution for 12 h at 37°C. The BrdU-labeled cells were then suspended in BrdU-free culture medium and lysed to extract apoptotic DNA fragments from the cytoplasm. The supernatant lysate (100μl) was transferred to 96-well, flat-bottom plate, which was pre-coated with anti-DNA antibody, incubated overnight at 4°C, and then denatured the DNA by microwave irradiation for 5 min on 500W power. Anti-BrdU antibody conjugated with peroxidase (100μl) was added and incubated overnight at 4°C. Finally, 3, 3', 5, 5'-tetramethylbenzidine (TMB) (100μl) was used as the substrate solution and the absorbance of the samples was measured at 450 nm using a microplate reader (HBS-1096A, DeTie Laboratory Equipment Co., Ltd., Nanjing, China). The rate of apoptosis is presented as the fold change of the absorbance of shRNA-β4 to shRNA-scramble-treated cells. Experiments were repeated three times.

### Gene set enrichment analysis (GSEA) and pathway relation network (Path-Net) analysis

Five hundred cases of PTC in TCGA cohort were stratified into high and low β4 expression groups by the median value of β4 mRNA expression level. GSEA (ver.3.0, http://www.broad.mit.edu/gsea/) was applied to identify gene sets enriched in the β4 high expression group. Gene sets were compiled from the Kyoto Encyclopedia of Genes and Genomes (KEGG). An enrichment score (ES) was calculated for gene sets, and then, a random permutation analysis was performed to estimate the significance level of ES and adjust for multiple hypothesis testing. We performed 1000 permutations and selected a false discovery rate (FDR) threshold <0.05 to designate statistically significant enrichment.

Path-Net analysis was further employed to reveal potential interactions among the significant pathways established from the above GSEA. Path-Net reflected the interaction features of the dysregulated pathways directly and systemically 33, 34. This network was built based on the interactions among the pathways in KEGG database and analyzed using Cytoscape software (ver.3.2, https://cytoscape.org/). We chose 45 significant pathways, including 25 upregulated pathways and 20 downregulated pathways, for Path-Net analysis and used “Degree” to evaluate the pathway interactions.

### Statistical analysis

All data were analyzed using SPSS Ver.22.0 (IBM Corporation, Armonk, NY, USA) and GraphPad Prism Ver.7.0 (GraphPad Software, Inc., La Jolla, CA, USA). Student's two-tailed *t*-test was used to compare the β4 mRNA expression levels between PTC and normal thyroid tissues in TCGA cohort. Correlation analysis between β4 expression levels and clinicopathologic characteristics was performed using a chi-square test (Pearson test and continuity correction test). The impacts of the clinicopathological factors on overall survival (OS) were evaluated using univariate Cox regression analyses. Multivariate Cox regression analyses were performed to determine independent prognostic factors based on the factors that were significant in the univariate Cox regression analyses. The Kaplan-Meier method was used to delineate survival curves. *In vitro* experimental data were compared between two groups using Student's two-tailed *t*-tests, and comparisons between three sets of data were analyzed using one-way ANOVA. The data were shown as the mean ± SEM and were obtained from three independent experiments. *P*<0.05 was considered statistically significant.

## Results

### Relationship between β4 mRNA expression and clinicopathological features of PTC

**Figure 1:**
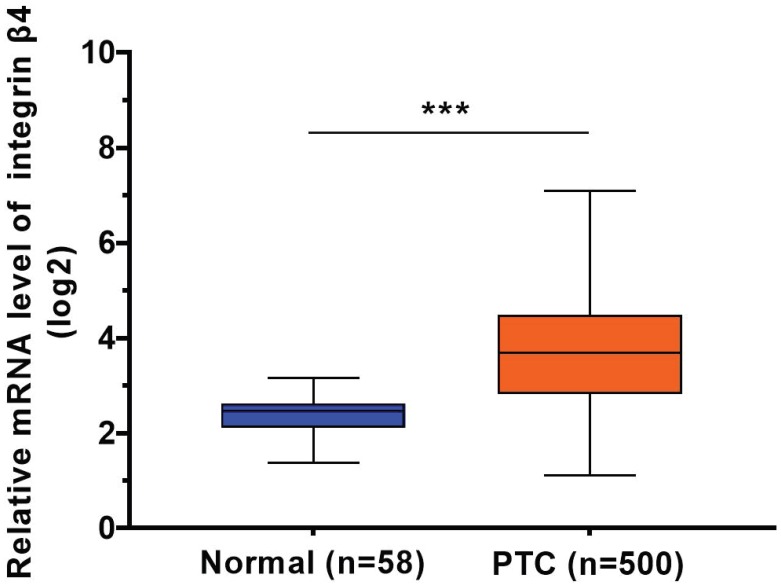
The relative mRNA expression level of integrin β4 in TCGA database, including 500 cases of papillary thyroid carcinoma (PTC) samples and 58 cases of normal thyroid samples. β4 mRNA expression was significantly upregulated in PTC. ^***^ *P*<0.001 versus normal thyroid tissue.

Using the data from TCGA cohort, we observed a significant increase in β4 mRNA expression in PTC tissue (n=500) compared to normal thyroid tissue (n=58) (*P*<0.001) (Figure 1). We categorized the 500 cases of PTC into low β4 expression (n=249) and high β4 expression (n=251) groups according to the median value of β4. Table 1 showed that high β4 expression was significantly associated with extrathyroid extension (*P*=0.044), lymph node metastasis (*P*<0.001), high T stage (*P*=0.01), and TNM stage (*P*=0.001). No statistical correlation was found with age, gender, lymphocytic thyroiditis or distant metastasis.

We found no significant difference in OS between the low and high β4 expression groups if the median β4 expression level was set as cut-off value (data not shown). However, if the 85th percentile of β4 expression level was set as the threshold, the OS of PTC patients was significantly different (*P*=0.029) between the two groups (Supplementary Figure S2). Patient age, extrathyroid extension, distant metastasis, high T stage, and high TNM stage were also significant factors associated with decreased OS. However, the Cox multivariate regression indicated that only extrathyroid extension and distant metastasis were independent predictors related to the unfavorable OS (Supplementary Table S2). These results suggested that overexpression of β was correlated with a low survival rate of PTC at the cut-off level of the 85th percentile; however, it was not an independent predictor.

### Relationship between β4 protein expression and histological features of PTC

**Figure 2:**
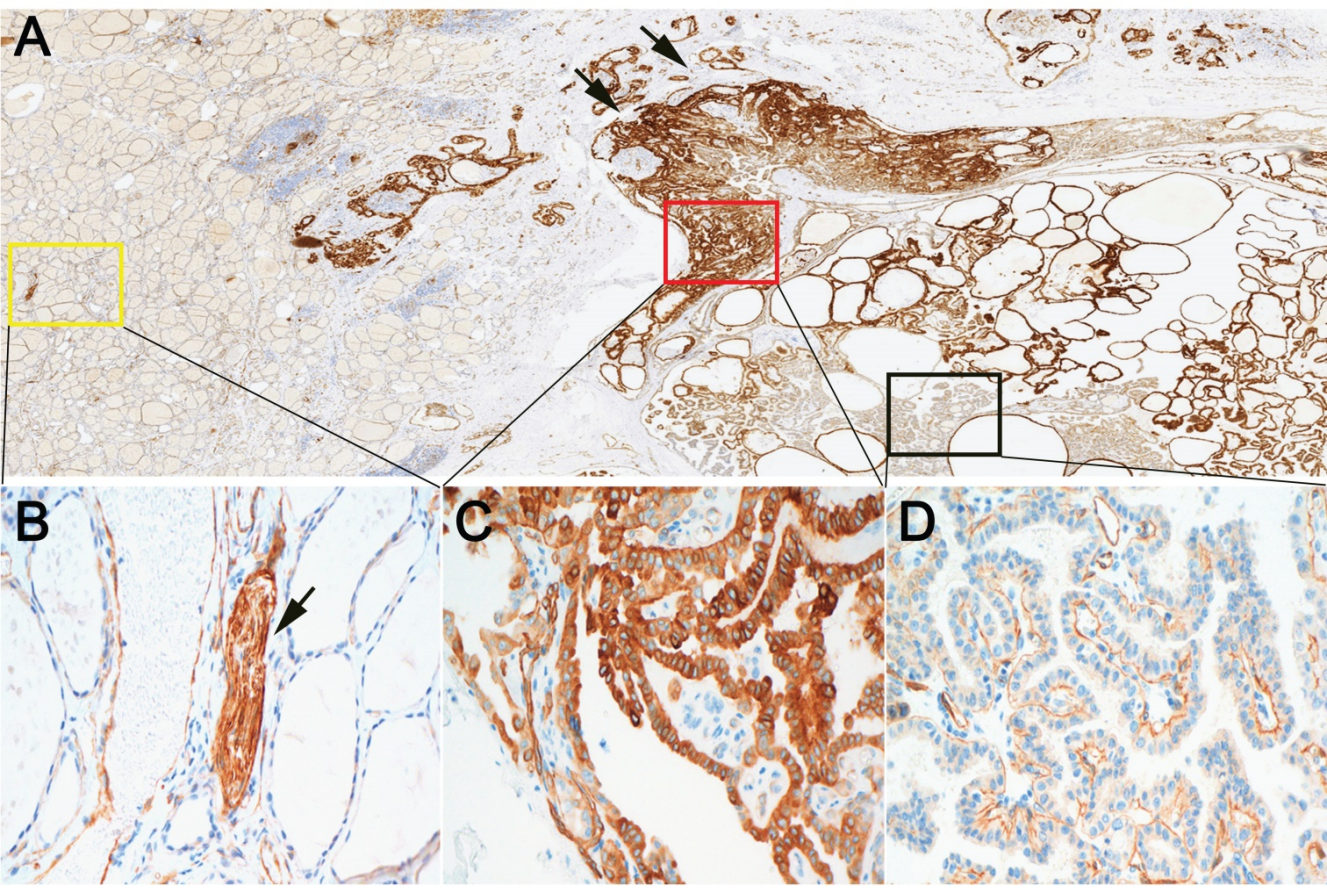
Expression of integrin β4 in papillary thyroid carcinoma (PTC) and surrounding noncancerous thyroid tissue by immunohistochemistry.** A:** At low power, β4 demonstrated positive immunoreactions with tumor cells of PTC. The staining intensity was heterogeneous inside the tumor and enhanced at the invasive frontier (arrow). **B:** The high power field view of yellow square marked in **A**. β4 exhibited negative or faint cytoplasmic staining in noncancerous follicular epithelia. In contrast, β4 was strongly expressed by the peripheral nerves (arrow). **C:** The high power field view of red square marked in **A**. Tumor cells in this area demonstrated equal or stronger cytoplasmic staining compared with that of peripheral nerves. It was defined as staining pattern 1. Tumor cells with staining pattern 1 were considered β4-overexpressing cells. **D:** The high power field view of black square marked in **A**. Tumor cells in this area demonstrated predominant basal membranous staining with little or no cytoplasmic staining. It was defined as staining pattern 2. Immunohistochemical stain (A-D). Original magnification: ×7 (A); ×400 (B-D).

We further evaluated the expression of β4 protein in 123 PTCs by immunohistochemistry. The tumor cells of PTC showed positive immunostaining of β4, while β4 was negatively expressed or very faintly stained in the cytoplasm of thyroid follicular cells in normal thyroid tissue (Figure 2A, 2B). Two staining pattern of β4 was observed in PTC. One was characterized by the strong cytoplasmic staining in tumor cells (pattern 1) (Figure 2C), and another was characterized by the predominant basal membranous staining with weak or no cytoplasmic staining in tumor cells (pattern 2) (Figure 2D). The tumor cells with staining pattern 1 were considered as β4-overexpressing cells. Thus, the PTC cases were categorized as low and high β4 expression group based on the staining scores of tumor cells with staining pattern 1 (see Materials and Methods).

**Figure 3:**
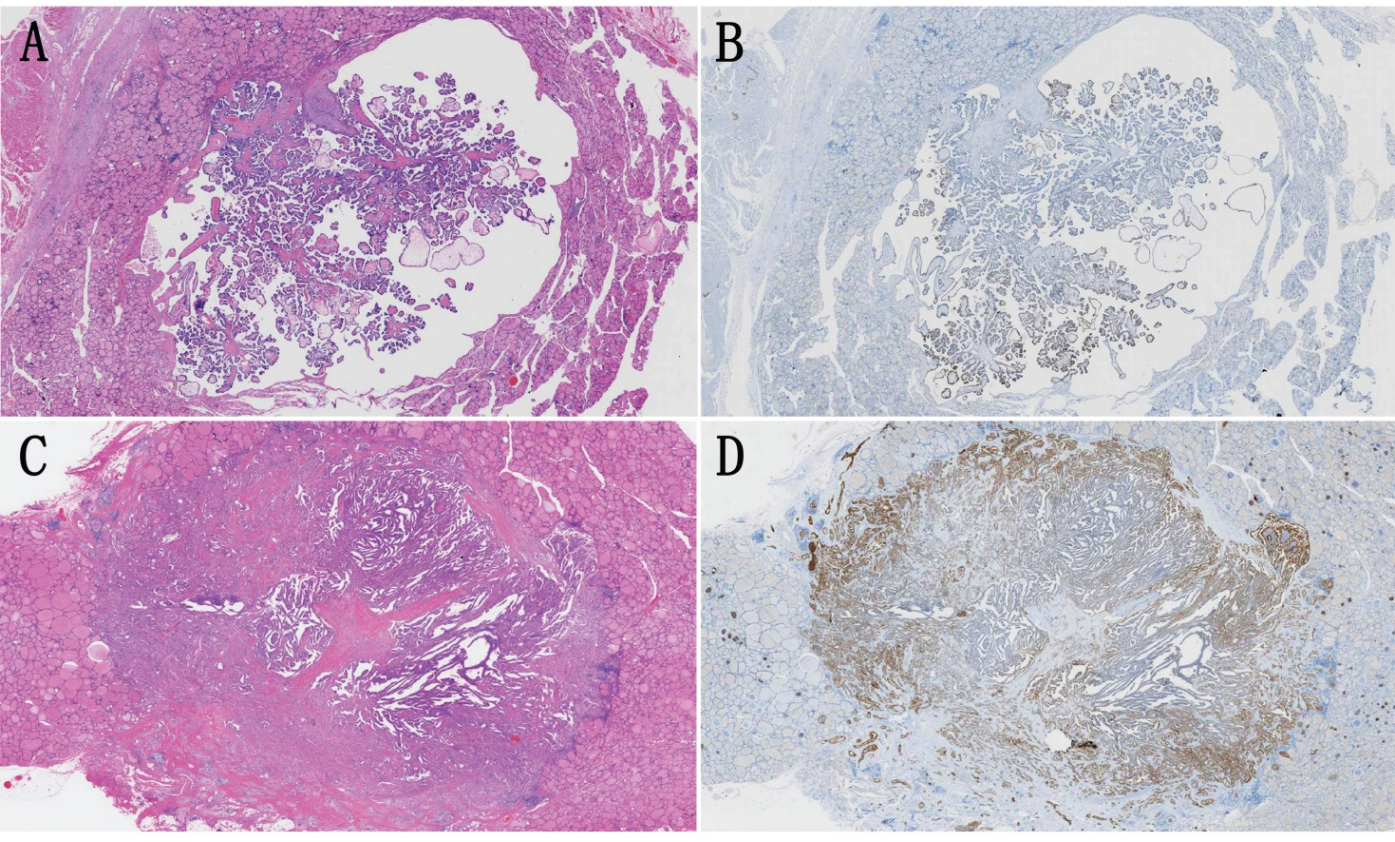
** A** and** B:** A representative case of papillary thyroid carcinoma (PTC) with well circumscribed growth pattern (WC-PTC), which was demarcated from surrounding noncancerous thyroid tissues by a thin capsule. The immunostaining of β4 was classified as low expression. **C** and** D:** A representative case of PTC with poorly circumscribed growth pattern (PC-PTC), which had an infiltrating border. The immunostaining of β4 was classified as high expression, and its expression was enhanced at the invasive frontier. Hematoxylin and eosin stain (A and C). Immunohistochemical stain (B and D). Original magnification: ×7 (A-D).

We noted that the β4 expression was frequently upregulated at the invasive frontier in PTC (Figure 2A), which prompted us to explore the β4 expression difference between WC-PTC and PC-PTC. The results revealed that β4 was significantly highly expressed in the PC-PTC group, in comparison with the WC-PTC group (*P*=0.015) (Table 2 and Figure 3). Previous studies have shown that compared with WC-PTC, PC-PTC exhibited an increased risk in lymph node metastasis and capsule invasion 23, 24, while the mechanism has not been fully illustrated. The increased β4 expression might be a potential contributor for the enhanced aggressiveness of PC-PTC.

Next, we evaluated the differential expression of β4 in 20 cases of the classic variant, 20 cases of the follicular variant, 20 cases of the oncocytic variant, 19 cases of the tall cell variant, 7 cases of the diffuse sclerosing variant and 12 cases of the solid variant of PTC. The results showed that there was no significant difference in β4 expression among these histological variants (*P*=0.55) (Table 2). Additionally, the BRAF^V600E^ mutation is thought to be associated with the high invasiveness of PTC, although there are still conflicting views 35, 36. Our results found no correlation between β4 expression and the BRAF^V600E^ mutation (*P*=0.195) (Table 2).

**Figure 4:**
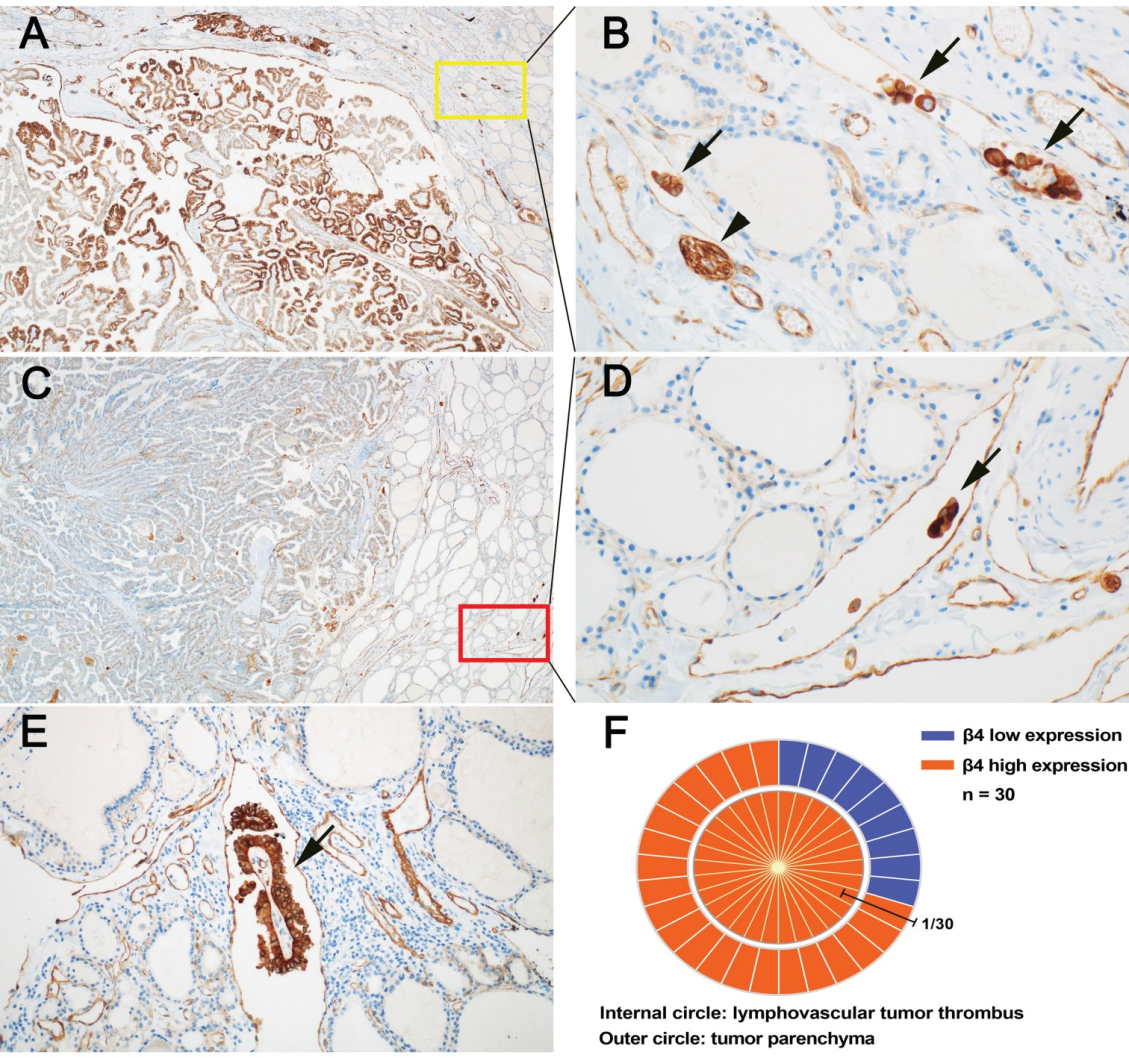
Expressional features of integrin β4 in papillary thyroid carcinoma (PTC) with lymphovascular tumor thrombus (LVT). **A** and** B:** In a representative case of PTC, the tumor parenchyma showed high expression of β4 (**A**), and the corresponding LVT (**B**) in yellow square**,** also demonstrated high expression of β4 (arrow). The expression intensity of LVT was approximately equal to that of peripheral nerves (arrowhead). **C** and** D**: In another case of PTC, the tumor parenchyma showed low expression of β4 (**C**), while the corresponding LVT (**D**) in red square**,** exhibited high expression of β4 (arrow). **E:** β4 was highly expressed in a focus of LVT with fibrovascular stroma (arrow). **F:** The schematic chart demonstrated the correlation of β4 expression between tumor parenchyma and intratumroal LVT. The outer circle represented the expression of β4 in the tumor parenchyma, of which 9 cases showed low expression (blue) and 21 cases exhibited high expression (red). The inner circle represented the expression of β4 in LVT. Notably, β4 was highly expressed in all the LVT. Immunohistochemical stain (A, B, C, D, and E). Original magnification: × 40 (A and C); × 200 (B and D); × 100 (E).

LVI has been shown to be a high-risk factor for persistent and recurrent events in PTC 37, 38. We found that the detection rate of LVI was significantly higher in the high β4 expression group than the low β4 expression group (*P*<0.001) (Table 2). It is suggested that increased expression of β4 may promote the occurrence of LVI. Particularly, we noted that in the 30 PTCs with LVI, all tumor thrombi exhibited high expression of β4 (Figure 4, B, D and E). Regarding their corresponding tumor parenchyma, 9 cases showed low β4 expression and 21 cases showed high expression (Figure 4, A, C and F). It indicates that regardless of the β4 expression status in the tumor parenchyma, once the tumor cells enter the circulatory system, the β4 expression will be upregulated or maintained at a high level. This phenomenon has not been reported before, and suggests that β4 might exert a control on the detached tumor cells through the alteration in expression level.

### The expression of β4 in PTC cell lines and the knockdown efficiency of β4-shRNA

**Figure 5:**
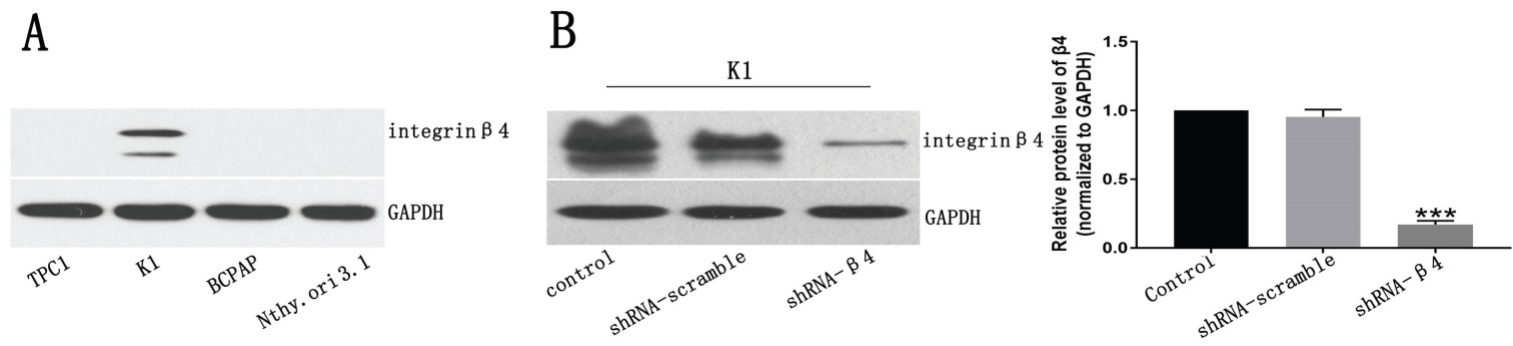
** A:** Western blot analysis for β4 protein expression in papillary thyroid carcinoma cell lines TPC1, K1, BCPAP and the human primary thyroid epithelial cell line Nthy-ori 3-1. β4 protein was detected only in K1 cells. **B:** The β4 expression in K1 cells was significantly reduced by shRNA-β4 treatment and the knockdown efficiency was around 80% compared with that of the control and shRNA-scramble groups. Data are expressed as the mean ± SEM, *n* = 3 (B). *** *P*<0.001 versus the control and shRNA-scramble groups.

We examined β4 protein expression in PTC cell lines, including TPC1, K1, BCPAP and the immortalized normal thyroid follicular epithelial cell line Nthy-ori 3-1, by Western blot analyses. The results indicated that its expression was detected only in K1 cells (Figure 5A). Thus, we chose K1 as the cell model and decreased its endogenous β4 expression by shRNA interference. The knockdown efficiency was approximately 80% as shown in Figure 5B.

### The knockdown of β4 inhibited K1 cells' migration, invasion, and motility

**Figure 6:**
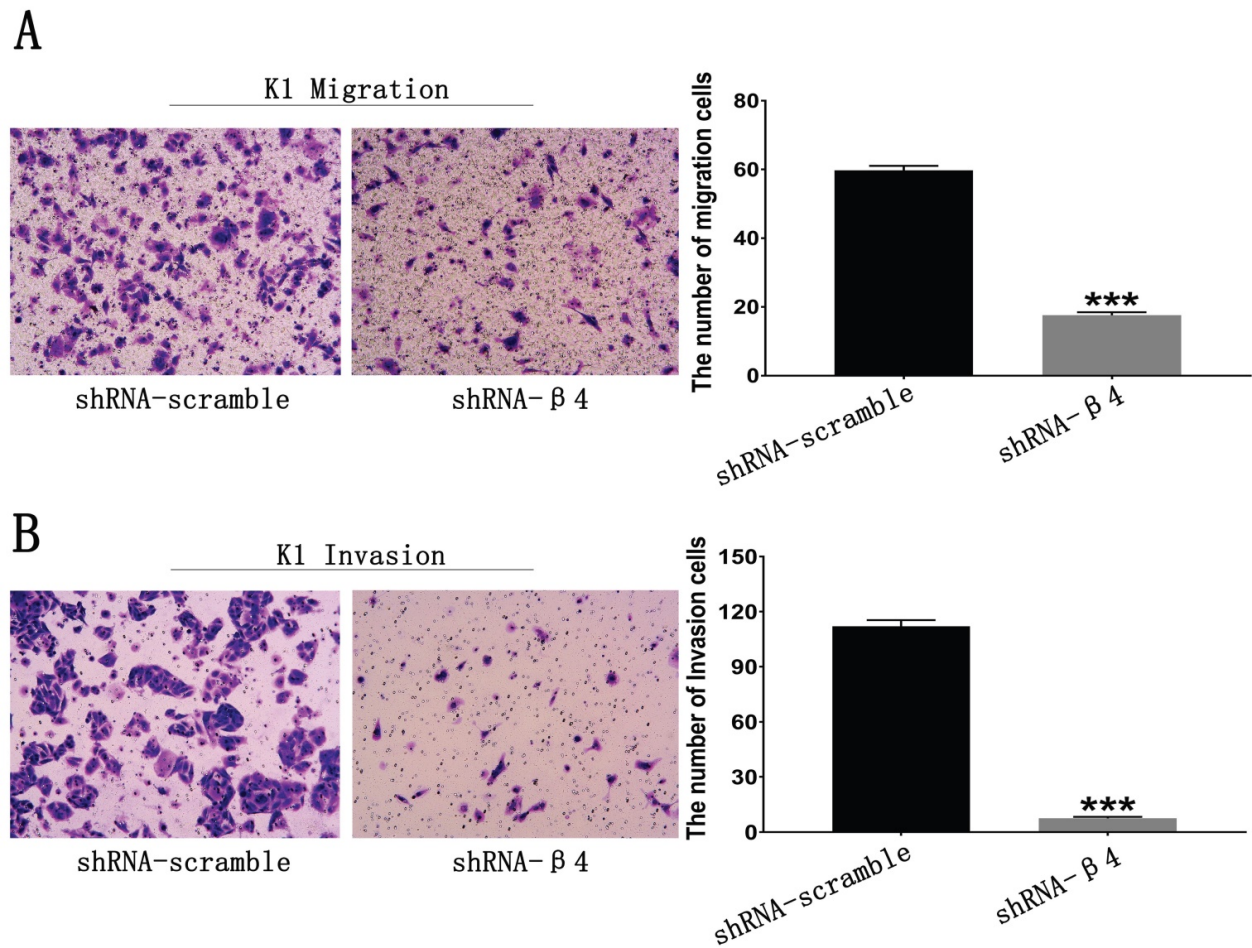
** A:** Transwell migration assay. The number of migrating K1 cells was significantly reduced after treatment with shRNA-β4 compared with that of the shRNA-scramble group. **B:** Transwell invasion assay. The number of invasive K1 cells was significantly reduced in cells treated with shRNA-β4 compared to the shRNA-scramble cells. Data are expressed as the mean ± SEM (A and B), *n* = 3 (A and B). *** *P*<0.001 versus shRNA-scramble.

**Figure 7:**
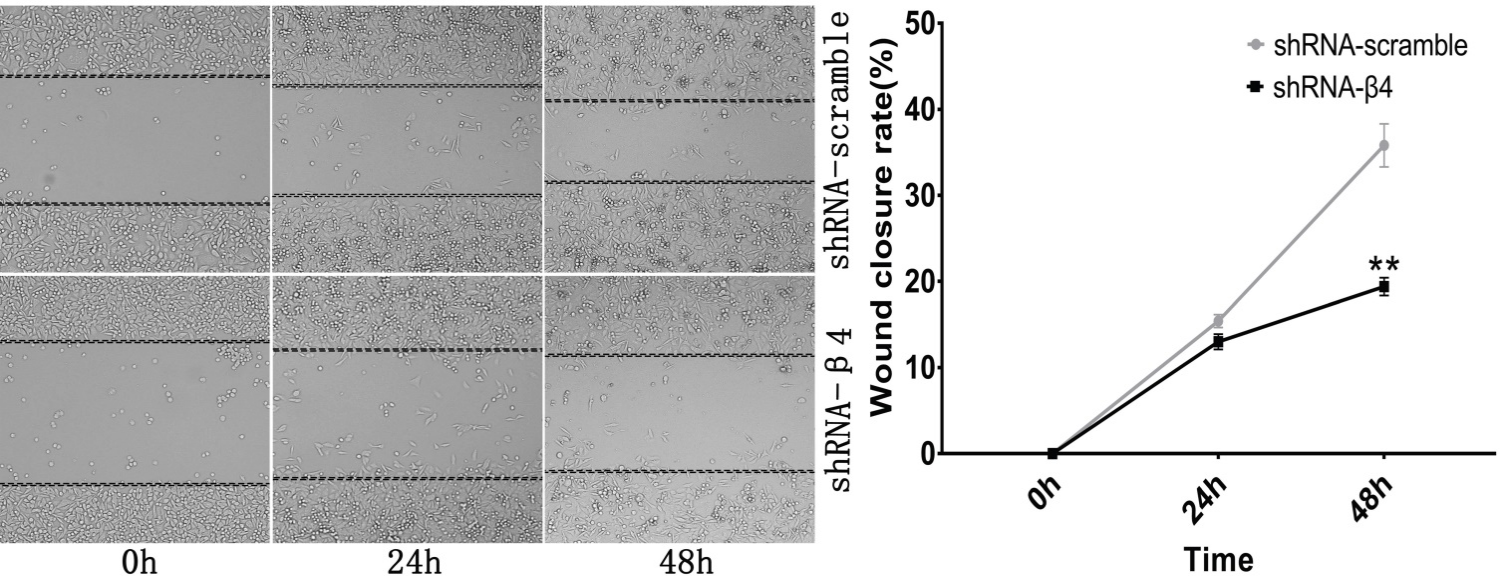
Wound healing assay. Photomicrographs were taken at the zero time point, 24h, and 48h to measure the ratio of the migrating distance relative to the initial wound distance. Wound closure significantly decreased at 48h after treatment with shRNA-β4. Data are expressed as the mean ± SEM. *n* = 3. ** *P*=0.003 versus shRNA-scramble.

The potential role of β4 on K1 cell's migration, invasion, and motility was further explored. As demonstrated in Figure 6A, the number of migrated K1 cells was significantly reduced after transfected with shRNA-β4 (*P*<0.001), compared with that of shRNA-scramble group. In addition, knockdown of β4 drastically inhibited K1 cell's invasion (*P*<0.001) (Figure 6B). Moreover, although the wound closure rate presented no obvious change at 24h (*P*=0.136), it was significantly reduced at 48h (*P*=0.003) in the shRNA-β4 group (Figure 7). These results indicated that β4 inactivation could suppress K1 cells' mobility.

### The knockdown of β4 reduced anchorage-independent growth and promoted anoikis

**Figure 8:**
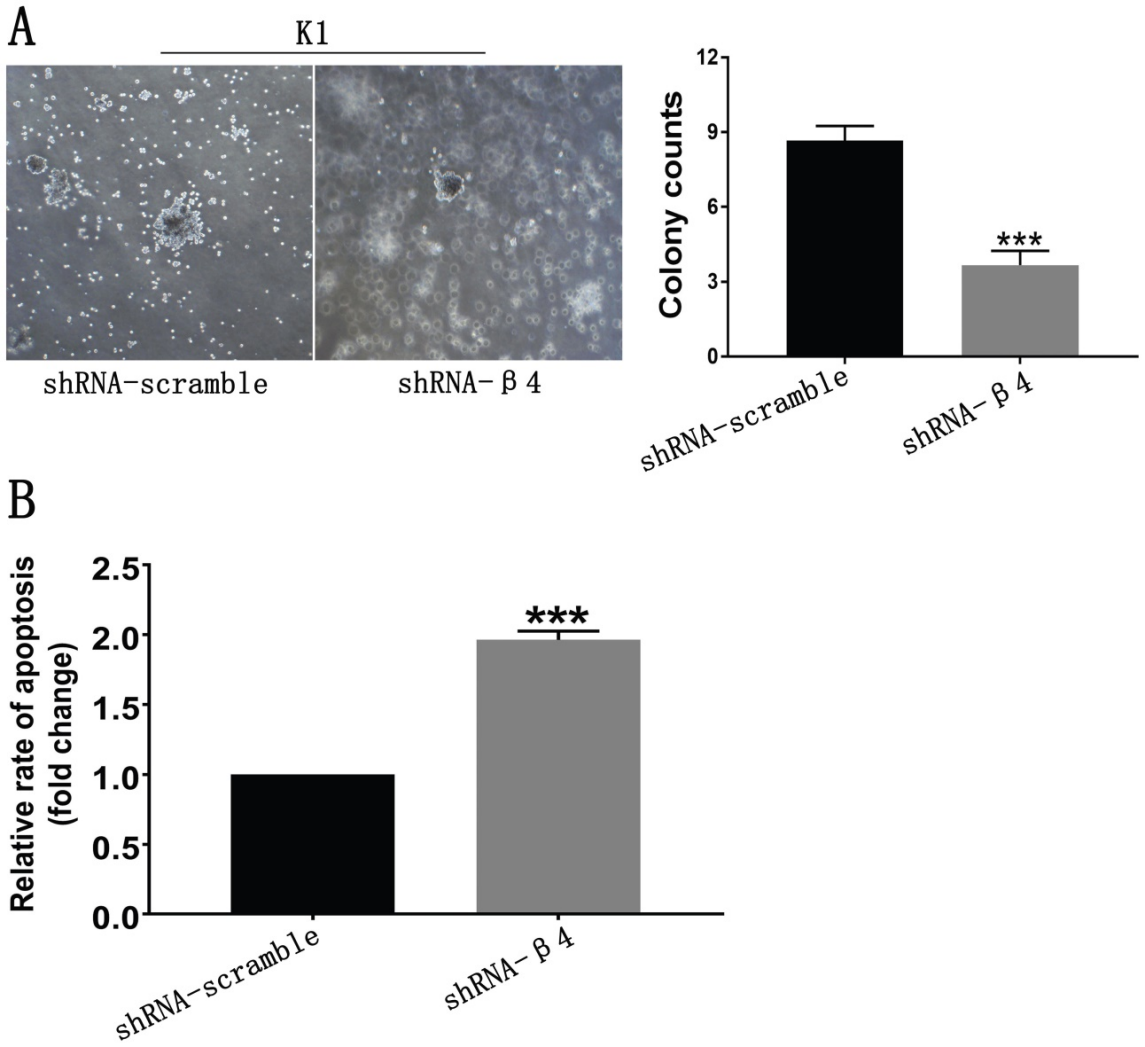
** A:** Soft agar assay**.** Representative bright-field images of colonies were captured. The shRNA-β4 group formed fewer colonies than the shRNA-scramble group. **B:** Anoikis analysis. Cell death was assessed by measuring cytoplasmic DNA fragmentation by ELISA. The rate of apoptosis was presented as the fold change of the absorbance (measured at 450 nm) of shRNA-β4 to shRNA-scramble-treated cells. Data are expressed as the mean ± SEM (A and B). *n* = 3 (A and B). *** P<0.001 versus shRNA-scramble.

Since anchorage-independent growth and resistance to anoikis are necessary for the distant spread of tumor cells 2, 39, the impact of β4 knockdown on those features was further tested. As shown in Figure 8A, K1 cells treated with shRNA-β4 formed fewer colonies than the control cells (*P*<0.001). In addition, they were more susceptible to anoikis in comparison with the control cells (*P*<0.001) (Figure 8B). The above data proved that increased expression of β4 could promote the neoplastic cells' survival and antagonize their apoptosis in PTC under the loss-of-adhesion condition.

### Metabolic and transductional pathways associated with high β4 expression

**Figure 9:**
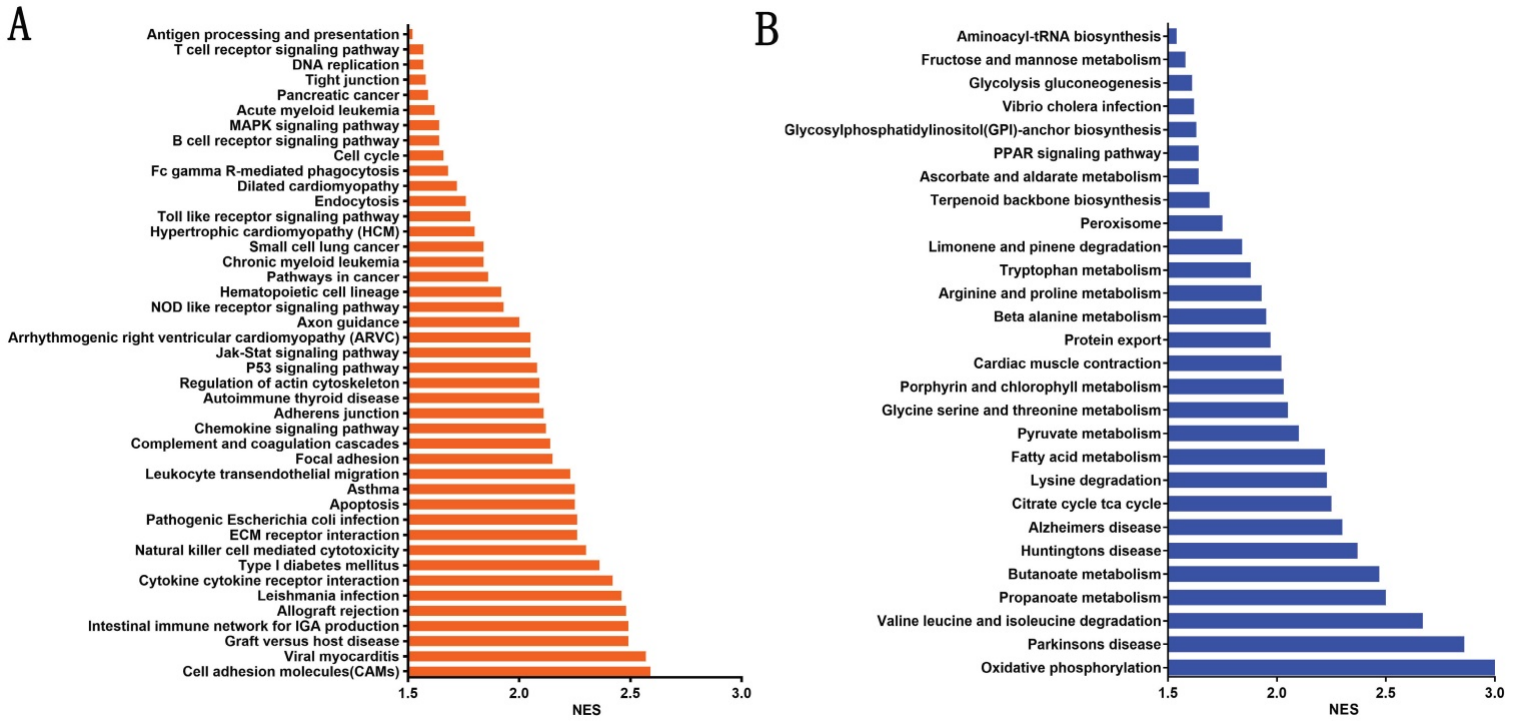
The significantly enriched metabolic and signaling pathways associated with high β4 expression in TCGA cohort. The enriched KEGG pathways were clustered by gene set enrichment analysis (GSEA). A false discovery rate (FDR) <0.05 was set to designate statistically significant enrichment, and the enriched pathways were sorted by the normalized enrichment score (NES). There were 43 significantly upregulated pathways (**A**) and 28 significantly downregulated pathways (**B**).

To evaluate the potential mechanisms by which β4 promotes PTC invasion and metastasis, we stratified PTC cases in TCGA cohort into β4 low and high expression groups according to the median value of β4 expression level, and explored the enriched pathways correlated with the high β4 expression. The GSEA analysis showed that there were 43 significantly upregulated KEGG pathways (Figure 9A), including cell adhesion molecules (CAMs), cytokine-cytokine receptor interactions and regulation of actin cytoskeleton. Those pathways were mainly involved in the regulation of cell-to-cell interactions and epithelial cell migration. In the meanwhile, 28 pathways including oxidative phosphorylation, propanoate metabolism, and the citrate cycle, were significantly down regulated (Figure 9B). Those pathways were mainly associated with the regulation of glucose, amino acid and fatty acid metabolism.

**Figure 10:**
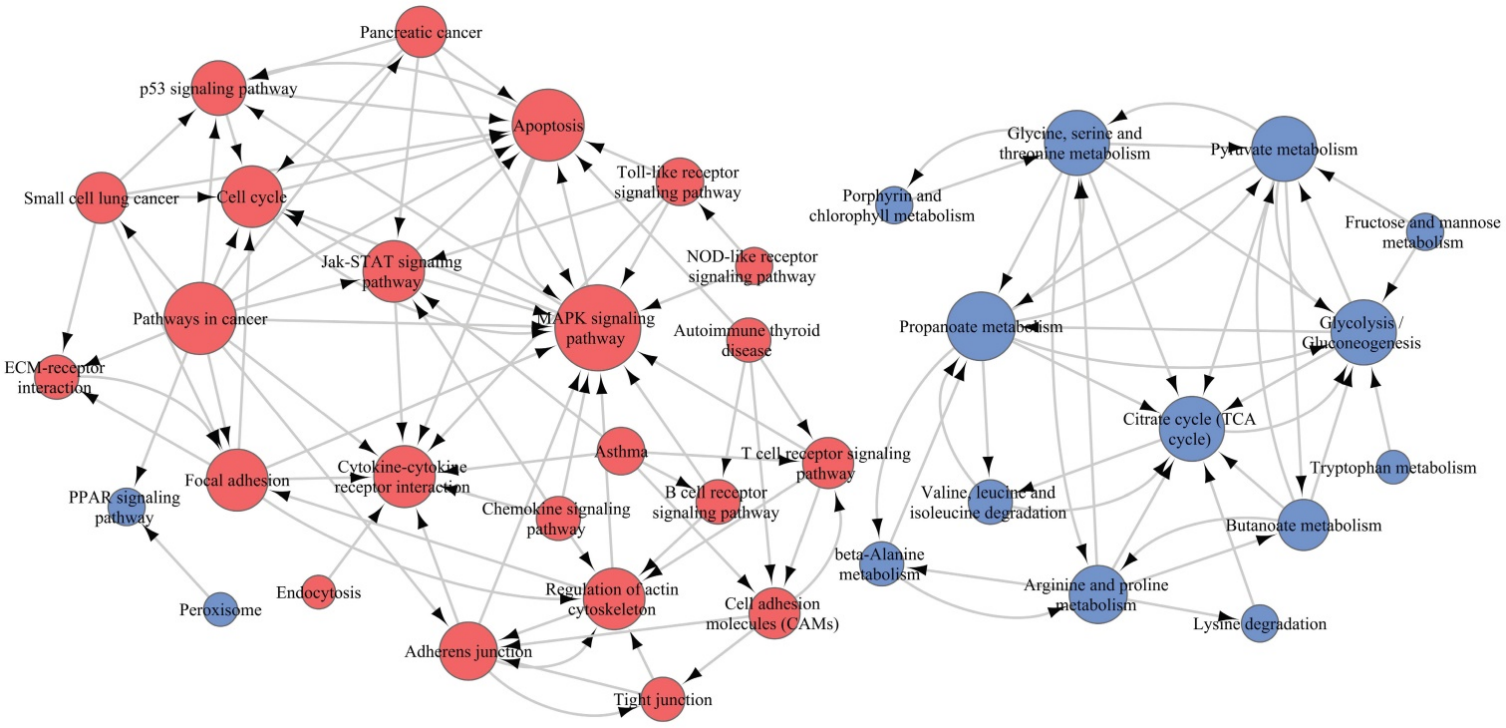
Pathway relation network (Path-Net) analysis. Forty-five significant pathways were chosen to build the Path-Net, and their interactions were evaluated by the value of Degree. As shown in the figure, there were 39 pathways with Degree≥1. Red dots represented significantly upregulated pathways, blue dots represented significantly downregulated pathways, and lines represented pathway's interactions. The diameter of the dots was positively correlated with the value of Degree.

To elucidate the interactions among these pathways, we selected 45 of 71 significant pathways, which were generally related to cell motivation, metabolism and signal transduction in malignancies, for Path-Net analysis. The results demonstrated that there were 39 pathways with a Degree ≥ 1 (Figure 10 and Supplementary Table S3). The MAPK pathway and propanoate metabolism were located in the network center among the upregulated and downregulated pathways, respectively. So the β4-associated increased invasiveness in PTC was more likely to be mediated by the comprehensive regulation on those pathways, specially centered on the nodes of MAPK pathway and propanoate metabolism.

## Discussion

Integrin β4 has been shown to promote the aggressiveness in some malignancies, such as invasive breast cancer and squamous cell carcinoma 14, 15, 17; however, its function in PTC has not been fully explored. Using the large cohort from TCGA, we demonstrated here that the β4 expression was significantly up-regulated in PTC compared with normal thyroid tissues. The high β4 expression was correlated with extensive extrathyroid extension, lymph node metastasis, high TNM stage, and compromised overall survival. These results overall indicated that increased β4 expression could boost the tumor's malignant progression.

Next, we explored the effect of β4 on the tumor's invasive process. The growth pattern of PTC has been reported to be associated with the tumor's invasiveness 22, 24, 27. WC-PTC has a low risk for thyroidal capsule invasion compared with that of PC-PTC 23. We found that β4 expression was significantly upregulated in PC-PTC, and the results strengthened the links between the high β4 expression and the increased invasive behavior in PTC. Moreover, through reducing the expression of β4 by RNA interference* in vitro*, we showed that the migration, invasiveness, and scratch repair ability of tumor cells were significantly reduced. Those *in vitro* data directly affirmed that β4 could promote the tumor's invasion, and further substantiate the findings from the TCGA and histological analyses.

Then, we explored the effect of β4 on the tumor's metastatic process. The presence of LVI has been considered a predictor for metastatic events 37, 38, 40. We demonstrated that in the high β4 expression group, the detection rate of LVI was significantly increased. Since β4 could enhance the tumor cells' invasiveness as shown above, it is reasonable to deduce that in high β4 expression group, more tumor cells infiltrate into the lymphatic and blood vessels, and thus lead to the increased incidence of LVI. Similarly, elevated β4 expression in non-small cell lung cancer has been reported to be associated with more venous invasion 41. In addition, β4 has been suggested to stimulate tumor cells' secretion for vascular endothelial growth factor 42, 43 and elicit increased angiogenesis 44, which further create a suitable milieu for the occurrence of LVI.

Circulating tumor thrombus, which is detached from the ECM, must overcome the effect of anoikis to achieve distant metastasis 45, 46. Our *in vitro* results demonstrated that β4 could enhance the tumor cells' anoikis resistance and anchorage-independent growth. Interestingly, we also observed that β4 was highly expressed in all tumor thrombi *in vivo*, which had no relations with the expression status in their corresponding tumor parenchyma. These findings suggest that the significance of high β4 expression in circulating tumor thrombi *in vivo* is to promote their survival under the anchorage-independent condition, and thus facilitate the occurrence of metastasis. Although the role of β4 in antagonizing anoikis has been recorded in breast cancer and liver cancer, these studies were all based on *in vitro* data 31, 47, 48. Our results for the first time provide the definite histological evidence on this process.

Considering some histological variants of PTC are associated with an aggressive phenotype 49-51, we further explored the correlations between histological variants of PTC and β4 expression status. Because WC-PTCs have distinctive clinicopathological features 22, 24, 27 and significantly decreased expression of β4 in comparison with PC-PTCs, we did not mix the 25 cases of WC-PTC (all of them belonging to the classical variant of PTC) with PC-PTC together during this process. So the histological variant analysis was established specifically on the cases of PC-PTC. The results showed there was no links between β4 expression and PTC variants. In addition, β4 expression was not associated with the BRAF^V600E^ mutant. We noted that β4 was generally highly expressed in the “hotspots” regions, such as the invasive fronts and intravascular tumor thrombi, while frequently expressed at low levels in the “quiescent” areas, such as the tumor's central region and metastatic tumor inside the lymph node parenchyma (data not shown). Thus, β4 expression in PTC is more likely dynamically altered to adapt to the tumor's progression rather than fixedly linked to an indicated genetic or histological variant.

To unravel the underlining mechanisms by which β4 promotes the tumor's aggressiveness, we performed the GSEA analysis and found that approximately 70 metabolic and signaling pathways were significantly dysregulated in the high β4 expression group. Among those upregulated pathways, the MAPK pathway was located in the network center. It has been proved that MAPK pathway plays a pivotal role in modulating the β4-dependent invasion and metastasis in invasive breast cancer and squamous cell carcinoma 15, 16, 52. In addition, the activated cell adhesion molecules (CAMs) and cytokine-cytokine receptor interaction pathways in PTC has been reported to boost lymph node metastasis 53. On the other hand, the majority of downregulated pathways are involved in the metabolic reprogramming process 2, 54. The attenuated glucose oxidative phosphorylation pathway has been shown to be a characteristic metabolic feature of PTC and is associated with the tumor's increased lymph node metastasis and high mortality 53, 55. Although most of those pathways need to be verified experimentally, the bioinformatics analysis provided a panoramic view of the regulation mechanism of β4.

Collectively, our study revealed that integrin β4 could promote invasion and antagonize anoikis of PTC, which are intrinsically linked to its upregulated expression in lymphovascular tumor thrombus and tumors with infiltrating growth pattern. Integrin β4 could be used as a predictive marker and a therapeutic target for highly aggressive PTC. Further studies will help clarify the interactions among the β4-associated pathways identified through the bioinformatics analysis.

## Supplementary Material

Supplementary figures and tables.

## Figures and Tables

**Table 1:** The relationship between integrin β4 mRNA expression and clinicopathological features in the TCGA cohort

Characteristic	Total, *n*(%)	β4 mRNA expression*, *n*(%)		*P* value
		Low	High	
**Sex**				0.963
Female	365 (73)	182 (73)	183 (73)	
Male	135 (27)	67 (27)	68 (27)	
**Age (years)**				0.175
<45	226 (45)	105 (42)	121 (48)	
≥ 45	274 (54)	144 (58)	130 (52)	
**Extrathyroid extension^†,§^**				**0.044**
None/minimal	463 (96)	231 (98)	232 (94)	
Moderate/Advanced	19 (4)	5 (2)	14 (6)	
**Lymphocytic thyroiditis^§^**				0.111
None	370 (84)	173 (81)	197 (87)	
Present	70 (16)	40 (19)	30 (13)	
**T stage^§^**				**0.010**
T1/T2	306 (61)	167 (67)	139 (56)	
T3/T4	192 (39)	82 (33)	110 (44)	
**N stage^§^**				**<0.001**
N0	227 (51)	138 (64)	89 (38)	
N1	223 (49)	78 (36)	145 (62)	
**M stage^§^**				0.995
M0	464 (98)	233 (98)	231 (98)	
M1	9 (2)	4 (2)	5 (2)	
**TNM stage^§^**				**0.001**
Ⅰ/Ⅱ	332(67)	183(74)	149(60)	
Ⅲ/Ⅳ	166(33)	65(26)	101(40)	

*The cases were stratified into high and low β4 expression group by the cut-off value set on median expression level of β4. †Extrathyroid extension is defined according to the 6th edition of TNM classification of thyroid carcinoma issued by American Joint Committee on Cancer (AJCC). Minimal extrathyroid extension refers to invasion to sternohyoid muscle or perithyroid soft tissue, and moderate/advanced extension refers to invasion to subcutaneous soft tissue, larynx, trachea, esophagus, or recurrent laryngeal nerve. §In the database of TCGA, there were 18 cases without the information on extrathyroid extension, 60 cases without the information on lymphocytic thyroiditis, 2 cases without the information on T stage, 50 cases without the information on N stage, 27 cases without the information on M stage, and 2 cases without the information on TNM stage.

**Table 2:** The relationship between the expression level of integrin β4 and histological features in papillary thyroid carcinoma

Characteristic	Total, *n* (%)	β4 protein expression, *n* (%)		*P* value
		Low	High	
**Growth pattern of PTC**				**0.015**
Well circumscribed	25(20)	20(28)	5(10)	
Poorly circumscribed	98(80)	52(72)	46(90)	
**Histologic variant of poorly circumscribed PTC**				0.55
Classical	20(20)	10(19)	10(22)	
Follicular	20(20)	14(27)	6(13)	
Oncocytic	20(20)	11(21)	9(20)	
Tall cell	19(19)	8(15)	11(24)	
Diffuse sclerosing	7(8)	4(8)	3(7)	
Solid	12(13)	5(10)	7(14)	
**BRAF^V600E^ mutation**				0.195
Positive	59(48)	31(43)	28(55)	
Negative	64(52)	41(57)	23(45)	
**Lymphovascular tumor thrombus**				**<0.001**
Present	30(24)	9(13)	21(41)	
Absent	93(76)	63(87)	30(59)	
