# Supplementary material for: Integrin β4 promotes invasion and anoikis resistance of papillary thyroid carcinoma and is consistently overexpressed in lymphovascular tumor thrombus
Source: J Cancer. 2019 Oct 21;10(26):6635–48. doi: 10.7150/jca.36125 (PMC6856897; doi:10.7150/jca.36125)
Supplement: Supplementary file 1 — Supplementary figures and tables. [file jcav10p6635s1.pdf]

Supplementary Table S1. Clinicopathological data of 123 patients with papillary thyroid carcinoma (PTC)

**Characteristic**	**Total, *n* (%）**	**Age (yr), mean±SD**	**Sex (female/male)**	**Tumor size (cm), mean±SD**
All cases of PTC	123（100）	36.63±12.02	84/39	1.60±0.76
Growth pattern of PTC				
Well circumscribed	25(20)	36.24±10.73	20/5	1.63±0.73
Poorly circumscribed	98(80)	36.72±12.37	64/34	1.59±0.78
Histologic variant of poorly circumscribed PTC				
Classical	20(20)	37.00±11.96	9/11	1.42±0.61
Follicular	20(20)	37.40±11.44	15/5	1.27±0.72
Oncocytic	20(20)	33.15±10.70	13/7	1.59±0.72
Tall cell	19(19)	37.94±14.21	13/6	1.64±0.68
Diffuse sclerosing	7(8)	29.00±8.12	6/1	2.64±1.11
Solid	12(13)	38.17±17.26	8/4	1.58±0.62
$BRAF^{V600E}$ mutation				
Positive	59(48)	35.69±11.90	40/19	1.62±0.83
Negative	64(52)	37.48±12.16	44/20	1.58±0.70
Lymphovascular tumor thrombus				
Present	30(24)	37.20±13.47	21/9	1.81±0.83
Absent	93(76)	36.44±11.58	63/30	1.53±0.73

Supplementary Table S2. Univariate and multivariate COX regression analysis of overall survival in papillary thyroid carcinoma in relation to β4 mRNA expression level and clinicopathological features

Characteristic	Total, *n*(%)	Univariate analysis		Multivariate analysis	
		**Hazard ratio (95% CI)**	***P*-value**	**Hazard ratio (95% CI)**	***P*-value**
Sex			0.195		
Female	365(73)	1			
male	135(27)	1.96(0.71-5.42)			
Age (years)			**0.034**		0.933
＜45	226(45)	1		1	
≥45	274(54)	79.76(1.38-4613.12)		4.69E+5（0.00-1.09E+138）	
Extrathyroid extension			**<0.001**		**0.027**
None/minimal	463(96)	1		1	
Moderate/Advanced	19(4)	9.86(3.40-28.56)		4.31(1.18-15.80)	
Lymphocytic thyroiditis			0.338		
None	370(84)	1			
Present	70(16)	0.04(0.00-29.29)			
T stage			**0.043**		0.938
T1/T2	306(61)	1		1	
T3/T4	192(39)	2.98(1.03-8.60)		0.94(0.20-4.40)	
N stage			0.534		
N0	227(51)	1			
N1	223(49)	1.43(0.47-4.38)			
M stage			**0.031**		**0.042**
M0	464(98)	1		1	
M1	9(2)	5.17(1.16-23.08)		5.72(1.07-30.57)	
TNM stage			**0.001**		0.916
I/II	332(67)	1		1	
III/IV	166(33)	7.22(2.32-22.42)		0.92(0.18-4.62)	
β4 mRNA expression*			**0.029**		0.277
Low	425（85）	1		1	
High	75（15）	3.09(1.12-8.51)		1.95(0.59-6.50)	

*The cases were stratified into high and low β4 expression group by the cut-off value set on 85th percentile expression level of β4.

Supplementary Table S3. The dysregulated pathways with degree≥1 in Path-Net analysis

Pathway name	Style	Degree	Indegree	Outdegree
MAPK signaling pathway	up	16	13	3
Apoptosis	up	12	9	3
Pathways in cancer	up	12	0	12
Propanoate metabolism	down	11	5	6
Citrate cycle (TCA cycle)	down	10	8	2
Glycolysis / Gluconeogenesis	down	10	7	3
Pyruvate metabolism	down	10	5	5
Glycine, serine and threonine metabolism	down	10	4	6
Cytokine-cytokine receptor interaction	up	9	9	0
Cell cycle	up	9	7	2
Regulation of actin cytoskeleton	up	9	6	3
Jak-STAT signaling pathway	up	9	5	4
Focal adhesion	up	9	4	5
Adherens junction	up	8	4	4
Arginine and proline metabolism	down	8	3	5
p53 signaling pathway	up	7	5	2
Cell adhesion molecules (CAMs)	up	6	3	3
T cell receptor signaling pathway	up	6	3	3
Butanoate metabolism	down	6	2	4
Pancreatic cancer	up	6	1	5
Small cell lung cancer	up	6	1	5
Toll-like receptor signaling pathway	up	5	1	4
Asthma	up	5	0	5
ECM-receptor interaction	up	4	3	1
B cell receptor signaling pathway	up	4	2	2
beta-Alanine metabolism	down	4	2	2
Tight junction	up	4	2	2
Valine, leucine and isoleucine degradation	down	4	2	2
Autoimmune thyroid disease	up	4	0	4
Chemokine signaling pathway	up	4	0	4
PPAR signaling pathway	down	2	2	0
Lysine degradation	down	2	1	1
Porphyrin and chlorophyll metabolism	down	2	1	1
Fructose and mannose metabolism	down	2	0	2
NOD-like receptor signaling pathway	up	2	0	2
Endocytosis	up	1	0	1
Peroxisome	down	1	0	1
Tryptophan metabolism	down	1	0	1

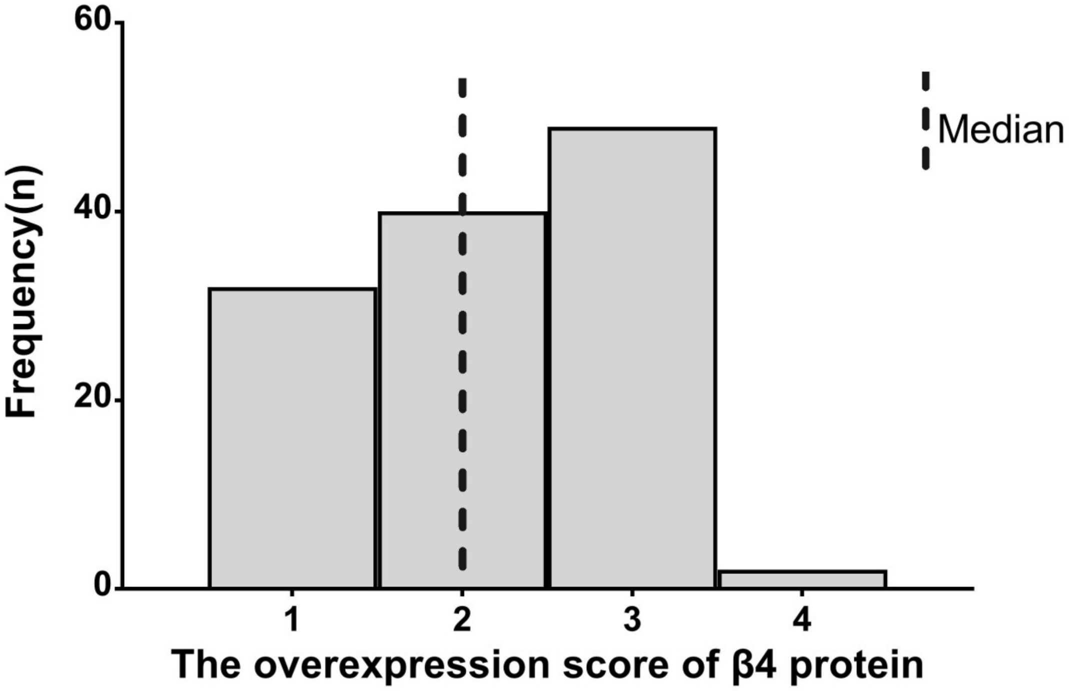

Supplementary Figure S1: The frequency histogram of β4 overexpression score established on 123 cases of papillary thyroid carcinoma. The number in the horizontal axis represented the overexpression score of β4, and the vertical axis indicated the frequency（case number）. The median overexpression score was 2 (indicated by dotted lines). Cases with overexpression score >2 were considered high β4 expression, while cases with overexpression score ≤2 were considered low β4 expression.

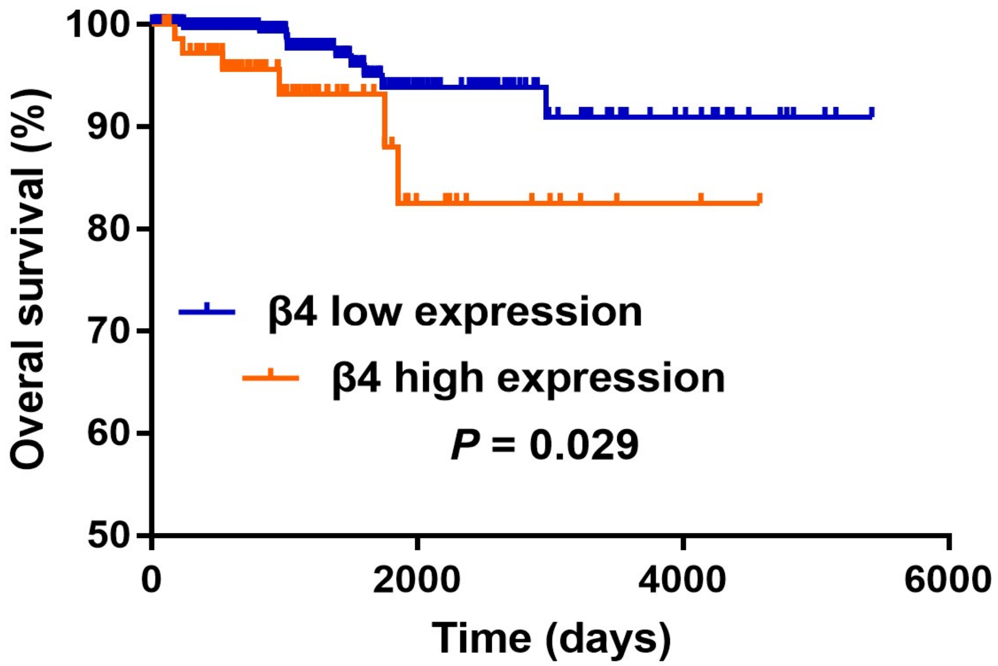

Supplementary Figure S2: Kaplan–Meier survival analysis demonstrated that high β4 mRNA expression in papillary thyroid carcinoma was significantly associated with poor overall survival in comparison with low β4 expression.
